# Lymphatic filariasis mapping by Immunochromatographic Test cards and baseline microfilaria survey prior to mass drug administration in Sierra Leone

**DOI:** 10.1186/1756-3305-5-10

**Published:** 2012-01-11

**Authors:** Joseph B Koroma, Momodu M Bangura, Mary H Hodges, Mohamed S Bah, Yaobi Zhang, Moses J Bockarie

**Affiliations:** 1 National Neglected Tropical Disease Control Program, Ministry of Health and Sanitation, Freetown, Sierra Leone; 2 Centre for Neglected Tropical Diseases, Liverpool School of Tropical Medicine, Liverpool, UK; 3 Helen Keller International, PO Box 369, Freetown, Sierra Leone; 4 Helen Keller International, Regional Office for Africa, Dakar, Senegal

## Abstract

**Background:**

National mapping of lymphatic filariasis (LF) was conducted using Immunochromatographic tests (ICT) in 2005 to determine endemicity and geographic spread of the disease. A baseline microfilaria survey was then conducted to determine LF prevalence and microfilaria intensity.

**Methods:**

In 2005 1,982 persons of 15 years and over from 14 health districts were selected and fingertip blood samples were tested with ICT cards. In 2007-8 blood samples were taken between 10 p.m. and 2 a.m. and examined for microfilaria (mf) from 9,288 persons from 16 sentinel sites representing each district and 2 additional sites for districts with populations over 500,000 (Bo and Kenema).

**Results:**

The overall LF prevalence by ICT cards was 21% (males 28%, females 15%). All districts had a prevalence of *Wuchereria bancrofti *antigen > 1%. Distribution of LF prevalence showed a strong spatial correlation pattern with high prevalence in a large area in the northeast gradually decreasing to a relatively low prevalence in the southwest coast. High prevalence was found in the northeast, Bombali (52%), Koinadugu (46%), Tonkolili (37%) and Kono (30%). Low prevalence was found in the southwest, Bonthe (3%) and Pujehun (4%). The mf prevalence was higher in the northeast: Bombali, 6.7%, Koinadugu 5.7%, Port Loko 4.4% and Kono 2.4%. Overall there was a significant difference in mf prevalence by gender: males 2.9%, females 1.8% (p = 0.0002) and within districts in Kailahun, Kono, Port Loko, Moyamba and Koinadugu (all p < 0.05). The mf prevalence was higher in people > 20 years (2.5%) than in people ≤ 20 years (1.7%) (p = 0.043). The overall arithmetic mean mf density was 50.30 mf/ml among mf-positive individuals and 1.19 mf/ml in the population examined which varied significantly between districts.

**Conclusions:**

The ICT results showed that LF was endemic nationwide and that preventive chemotherapy (PCT) was justified across the country. Both the ICT and microfilaraemia surveys found that prevalence was greater in males than females. The increase in microfilaraemia prevalence by age was evident when grouped as ≤ 20 versus > 20 years demonstrating early exposure. Baseline LF microfilaria load will be used to monitor PCT program progress.

## Background

Lymphatic filariasis (LF) is a chronic, debilitating disease that affects people in tropical and subtropical areas of Asia, Africa, the Western Pacific and some areas of the Americas caused by the parasites *Wuchereria bancrofti *or *Brugia malayi *and *B. timori*, transmitted by *Culex, Anopheles *and other mosquitoes [1-4]. An estimated 90% of all LF cases worldwide and all cases in Africa are infections with the parasite *W. bancrofti*. The main vectors in West Africa are the *Anopheles *mosquitoes [5]. Over 120 million people in over 80 countries worldwide in the tropics and subtropics and over 40 million people in Africa are infected with the parasite [6,7]. Bancroftian filariasis, which is prevalent in Africa, is endemic in rural as well as urban communities thriving within poor communities [5,8,9].

LF has a wide range of clinical manifestation from acute attacks of filarial fever, chronic conditions such as hydroceles, lymphoedema, elephantiasis of limbs, and enlarged breasts, to kidney damage, thus causing great morbidity and disability for those affected [10]. Filariasis is one of the most common causes of permanent disability worldwide creating the highest disease burden in terms of DALYs among tropical diseases [7]. Those affected also suffer psychosocial stigmatization and economic suffering as it can lead to job loss or inability to work. The disease is therefore a major cause of poverty as it creates economic burden for those affected, their dependants, their communities and the country as a whole [8,9,11-13]. In 1993 the International Task Force on Disease Eradication identified LF as one of six diseases that could be eradicated, which led the World Health Assembly in 1997 to pass resolution WHA 50.29 calling for the elimination of LF as a public health problem in the world by 2020. In 1998, the Global Alliance to Eliminate LF was formed to support LF elimination programmes in endemic countries and World Health Organization (WHO) launched a Global Programme for the Elimination of LF as a result [2,3,13-15].

Circulating microfilariae (mf) are responsible for transmission, therefore transmission can be broken/interrupted by reducing the number of people with microfilaraemia within affected communities through annual mass drug administration (MDA) for 4-6 years to ≥ 80% of the entire at-risk population which can reduce mf to zero or close to zero [16,17]. Before MDA is started in a country, implementation units to be targeted should be determined through a rapid assessment study and also baseline data on LF mf level should be obtained to monitor effectiveness of MDA. There are already countries that have succeeded in eliminating the disease (Cape Verde, China, Costa Rica, Solomon Islands, South Korea, Suriname, and Trinidad and Tobago) using a combination of strategies that include vector control and single annual doses of 2-drug treatments (albendazole together with ivermectin or diethylcarbamazine) [16,17]. The current preferred strategy for LF elimination recommended by WHO is the preventive chemotherapy using the available drugs [18,19]. The global LF elimination programme has been strengthened by donation of albendazole by GlaxoSmithKline and continued donation of ivermectin by Merck & Co [20].

In Sierra Leone, reports from health facilities indicated endemicity of LF in all districts, and in 1996, a study in Moyamba district using the thick blood film method showed mf prevalence: 10.2% with 36.5% clinical manifestation (26.6% hydroceles and 9.4% lymphoedema/elephantiasis of the lower extremities) [21]. In 2004, a country profile of communicable diseases in Sierra Leone developed by WHO, quoting an anonymous 1999 study in 55 sites including the capital Freetown, showed that 14.5% of people tested for circulating filarial antigen of *W. bancrofti *were positive [22]. The Northern Province had the highest prevalence (19.6%), followed by Western Area (12.8%), Eastern Province (12.7%) and Southern Province (10.9%) [22]. Infection was noted in children, and this indicated ongoing transmission and infections acquired early in life.

In preparation for the national LF elimination programme, national mapping was conducted in 2005 using immunochromatographic test (ICT) cards. The national LF elimination programme started when the Ministry of Health and Sanitation (MoHS), Sierra Leone in consultation with WHO decided to conduct the integrated management of onchocerciasis, LF, schistosomiasis, soil-transmitted helminthes and trachoma, and the existing National Onchocerciasis Control Programme became National Neglected Tropical Disease Control Programme (NTDCP) in 2006 [23]. Based on the mapping results, the implementation units (districts) for LF MDA were determined and national baseline data on mf level were collected pre-MDA. The current paper presents the distribution and the level of infection of LF in Sierra Leone which formed the base for the national LF elimination programme.

## Methods

### Ethics statement

The studies were conducted by the National NTDCP of the MoHS, Sierra Leone. Ethical approval for data collection was obtained from the Ethics Committee of the MoHS and upon arrival at the randomly selected communities the investigating team met with the community leaders and explained the nature of their work. All participants aged 15 years or above in each site were eligible for inclusion without discrimination on gender, social status, religion or ethnicity. People participated in the studies after informed consent was verbally obtained and recorded by the team leader, as literacy rates are low in Sierra Leone. Data collection was conducted such that participants remained anonymous during data entry and analysis. No individual identity can be revealed upon publication.

### National mapping of LF with ICT cards in 2005

Although previous studies and clinical records indicated that LF was prevalent particularly in the north, detailed data on distribution and level of risk throughout the country was not available, and all districts including the Rural Western Area both Rural (RWA) and Urban Western Area (UWA) were included in the mapping. Thirty-four (34) communities were randomly selected from all districts in consultation with WHO/AFRO with each district having at least one community selected. Participants who were 15 years of age or above were selected for the antigenaemia study using ICT cards [24]. During the survey ICT cards were kept overnight at 8°C in district cold rooms for the expanded programme on immunization and during the day the cards were transported in cold boxes and vaccine carriers with ice packs. The left index finger was pricked with a sterile lancet after cleaning with cotton wool soaked with spirit and 100 μl blood collected and applied straight to the ICT cards. The survey teams were trained to read the results of the ICT cards at exactly 10 minutes after application, according to the manufacturer's instructions. No late reading of ICT cards was reported by the survey teams. Due to limited resources and the high sensitivity and specificity of the ICT [25,26], fifty (50) persons were sampled in each randomly selected community and if a positive case was identified the sampling was complete and the teams moved on to the next site. If in the first 50 samples there was no positive case found, a further 50 persons were sampled bringing the total to 100 per site [26]. Training of technicians in the use of the ICT cards to detect *W. bancrofti *antigen and data recording took place in Makeni and York Village, RWA. Three teams of three technicians (2 for specimen collection and 1 for reading and recording of results) worked with a village volunteer (usually a school teacher) who served as registrar. Data included name, sex, village, chiefdom and district. A total of 1,982 people were tested; males 904 (45.6%) and females 1,078 (54.4%).

### Baseline microfilaraemia data collection before MDA

Sampling was conducted in accordance with WHO guidelines [26] of two sentinel sites per implementation unit (district for Sierra Leone) with a population of one million people. Ten of the fourteen health districts of Sierra Leone have a population below 500,000 and one sentinel site per district was selected representing a population ≤ 500,000. One sentinel site each was selected for RWA (232,294) and UWA (901,953). Bo district (Bo Town: Bo District, 222,561: 347,610) and Kenema district (Kenema Town: Kenema District, 188,869: 377,067) had population above 500,000 and two sentinel sites were selected in these two districts, making a total of 16 sites. Communities/villages that showed the highest ICT prevalence in each district in 2005 were selected as mf sentinel sites [26]. The survey was performed in two phases according to funding availability: Bombali, Koinadugu, Kambia, Kono, Kailahun and Pujehun in 2007 and Tonkolili, Port Loko, Kenema, Bonthe, Moyamba, Bo, RWA and UWA in 2008. Pre-sensitization was carried out before the survey in each site. Five hundred participants of 15 years of age or above were recruited per site. In sites with less than 500 people selected, extra participants were recruited in neighbouring villages. To ensure the standardization of activities and data, two day practical training was performed before the study started for all technicians. Fingertip blood was collected between 10 p.m. and 2 a.m. from each volunteer. A 60 μl blood sample was collected, smeared gently and uniformly in a circular shape and allowed to air dry at room temperature for 12-24 hours. The following day the dried smear was dehaemoglobinized by flooding with distilled water for 3-5 minutes, air dried again, fixed with methanol for 30-60 seconds, stained with Giemsa for 10 minutes then examined for mf under a light microscope by experienced examiners. Positive findings of mf were recorded and individual mf density of infection was calculated and expressed as the number of mf per ml of blood (mf/ml) [26-29]. A total of 9,288 night blood samples were examined. The mean age (± standard deviation) of the subjects examined was 37.7 ± 17.04 years (males: 37.27 ± 17.6, females: 38.12 ± 16.5). For quality control, all positive slides and 10% of the negative slides were preserved and examined by an experienced researcher.

### Statistical analysis

Results were entered into Epi-Info version 3.5.2 and analyzed in SPSS (IBM, Version 19). Prevalence of positive circulating LF antigen or microfilaraemia was calculated. The 95% confidence intervals (CIs) for prevalence were calculated using the Wilson score method without continuity correction [30]. The arithmetic mean mf density of infection with 95% CIs was calculated using the total population examined and the positive samples only [26]. Chi-square test was used to compare the differences in prevalence and Kruskal-Wallis test was used to compare the differences in mf density. Correlation analysis was conducted for the two sets of data (ICT and microfilaraemia prevalences) and the significance of the correlation tested [31]. The coordinates of each sample site were recorded using hand-held units of global positioning system (site coordinates available upon request). Spatial analysis of the LF antigenaemia prevalence (ICT card data) was conducted using the kriging method in the Geostatistical Analyst Extension of ArcGIS version 10 (ESRI, Redlands, USA). Spatially smoothed contour maps of the interpolated prevalence of antigenaemia and the predictive probability for the ICT prevalence of greater than 1% were produced [32].

## Results

### Distribution of lymphatic filariasis in Sierra Leone

**Figure 1:**
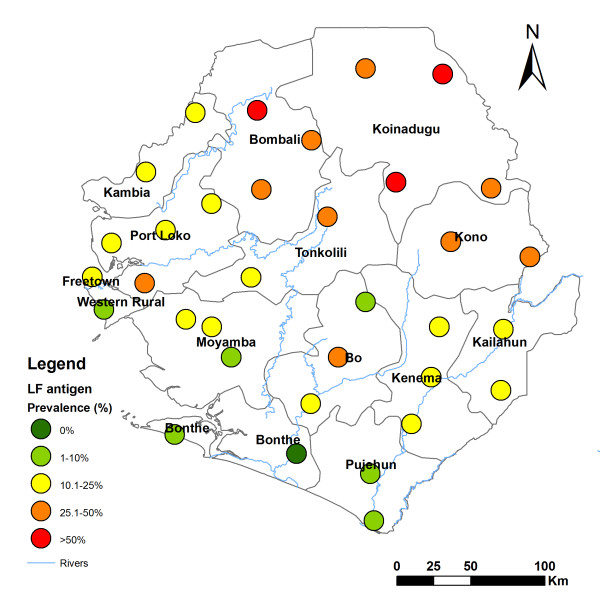
**Geographical distribution of lymphatic filariasis point prevalence by circulating antigen detection with ICT cards according to survey locations in Sierra Leone**.

Table 1 summarizes the results of the survey using ICT cards for each district. All the districts of Sierra Leone were found to be endemic for LF with a prevalence of ICT positive tests ≥ 1%. Point prevalence for each survey site was shown in Figure 1. Among 34 sites surveyed, only one site in Bonthe district was shown to be negative. The median prevalence across the 34 sites was 20% ranging from 0% to 68% (inter-quantile range: 11.7-31%). Overall ICT positive prevalence was 20.8%. High prevalence was found in the northeast part of the country (Bombali 52%, Koinadugu 46%, Tonkolili 37% and Kono 30%). Relatively low prevalence was found in the southwest coastal districts (Bonthe 3.1% and Pujehun 4.4%).

**Table 1:** Crude LF prevalence with antigen detection and microfilaraemia tests by district, sex and age group in Sierra Leone

	No of persons tested by ICT cards	Percentage prevalence of antigen positives (95% CI)	No of persons examined for Mf	Percentage prevalence of Mf positives (95% CI)	Population Mf density (mf/ml) (95% CI)	Positive-only Mf density (mf/ml) (95% CI)
**Overall**	1982	20.8 (19.1 - 22.7)	9288	2.4 (2.1 - 2.7)	1.19 (0.90 - 1.48)	50.3 (39.89 - 60.71)
***By district***						
Bo	173	15.0 (10.5 - 21.1)	1005	2.0 (1.3 - 3.1)	1.97 (0.84 - 3.11)	99.17 (58.32 - 140.01)
Bombali	150	52 (44.1 - 59.8)	830	6.9 (5.3 - 8.8)	1.93 (1.28 - 2.57)	28.07 (21.70 - 34.44)
Bonthe	160	3.1 (1.3 - 7.1)	504	1.2 (0.6 - 2.6)	0.83 (0.02 - 1.63)	69.44 (13.68 - 125.21)
Kailahun	110	19.1 (12.8 - 27.4)	624	2.6 (1.6 - 4.1)	2.08 (0.00 - 4.89)	81.25 (0.00 - 195.58)
Kambia	110	15.5 (9.9 - 23.4)	619	2.1 (1.2 - 3.6)	0.97 (0.23 - 1.71)	46.15 (17.04 - 75.27)
Kenema	180	13.3 (9.1 - 19.1)	1016	0.6 (0.3 - 1.3)	0.34 (0.00 - 0.70)	58.33 (4.42 - 112.24)
Koinadugu	200	46 (39.2 - 52.9)	636	5.7 (4.1 - 7.7)	1.99 (0.95 - 3.04)	35.19 (19.83 - 50.54)
Kono	100	30 (21.9 - 39.6)	875	2.4 (1.6 - 3.6)	1.11 (0.37 - 1.84)	46.03 (20.09 - 71.97)
Moyamba	200	10.5 (7.0 - 15.5)	500	1 (0.4 - 2.3)	0.67 (0.00 - 1.36)	66.67 (6.33 - 127.00)
Port Loko	210	20.5 (15.6 - 26.4)	500	4.4 (2.9 - 6.6)	3.53 (1.48 - 5.59)	80.30 (44.49 - 116.12)
Pujehun	160	4.4 (2.1 - 8.8)	624	0 (0 - 0.6)	-	-
Tonkolili	100	37 (28.2 - 46.8)	500	2.4 (1.4 - 4.2)	0.63 (0.24 - 1.03)	26.39 (17.99 - 34.79)
WA Rural	69	7.3 (3.1 - 15.9)	500	1.2 (0.6 - 2.6)	0.33 (0.01 - 0.65)	27.78 (6.60 - 48.96)
WA Urban	60	11.7 (5.8 - 22.2)	555	0 (0 - 0.7)	-	-
						
***By sex***						
Male	904	27.5 (24.7 - 30.6)	4335	3.0 (2.6 - 3.6)	1.66 (1.11 - 2.20)	54.42 (38.81 - 70.02)
Female	1078	15.2 (13.2 - 17.5)	4953	1.8 (1.4 - 2.2)	0.78 (0.52 - 1.04))	44.13 (32.50 - 55.76)
						
***By age group (yrs)***						
15-20	-	-	1873	1.8 (1.3 - 2.5)	0.78 (0.38 - 1.19)	44.44 (26.37 - 62.52)
21-30	-	-	2019	2.6 (2.0 - 3.4)	1.77 (0.73 - 2.80)	68.59 (31.79 - 105.39)
31-40	-	-	1830	2.4 (1.8 - 3.2)	1.01 (0.55 - 1.47)	43.02 (27.55 - 58.50)
41-50	-	-	1404	3.4 (2.5 - 4.4)	1.60 (0.94 - 2.27)	47.87 (32.75 - 62.99)
> 50	-	-	2162	2.1 (1.6 - 2.8)	0.89 (0.49 - 1.30)	42.96 (27.40 - 58.53)

There were significantly more positive ICT tests in males (27.54%, 95% confidence interval (CI): 24.7-30.6%) than in females (15.2%, 95% CI: 13.2-17.5%) (*p *< 0.00001). Detailed analysis of prevalence among different age groups was not carried out as detailed age information was not recorded for the ICT card survey.

### Microfilaraemia prevalence and density

Overall 9,288 night blood samples, male 4,335 (46.7%) and female 4,953 (53.3%), were examined for mf as shown in Table 1. There were less than 5% false positives and no false negative slides. All positive slides were reexamined and 3 were redefined as artifacts. No mf of *Mansonella perstans *was detected.

In total, 220 persons (2.4%, 95% CI: 2.1-2.7%) had a positive blood smear and there was significantly higher mf prevalence in males 3.0% (95% CI: 2.6-3.6%) versus females 1.8% (95% CI: 1.4-2.2%) (p = 0.0002). Age distribution of the mf prevalence is also shown in Table 1. There was a significant difference in mf prevalence among age groups with higher prevalence in persons of 41-50 years (p = 0.041).

**Figure 2:**
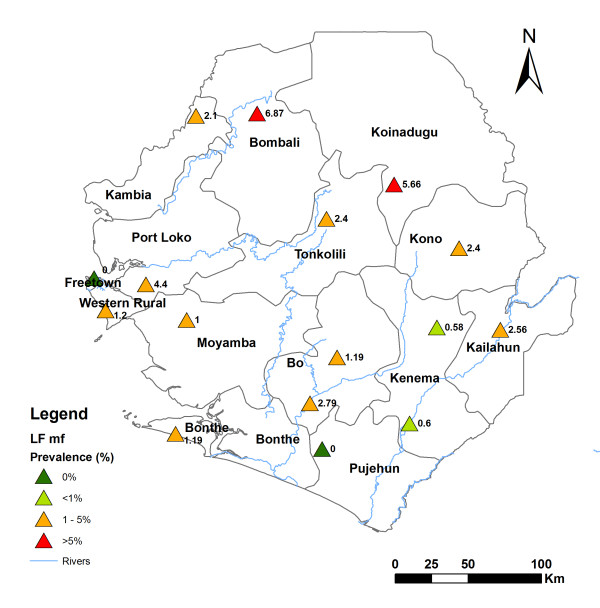
**Geographical distribution of lymphatic filariasis point prevalence by mf detection and point mf density according to survey locations in Sierra Leone**.

The point prevalence of microfilaraemia for each site is shown in Figure 2. There was a significant correlation between the mf prevalence and the ICT card prevalence (r = 0.86, p < 0.05). In line with the ICT results, the mf prevalence (95% CI) was higher in the northeast part of the country: Bombali, 6.9% (5.3-8.8%), Koinadugu 5.7% (4.1-7.7%), Port Loko 4.4% (2.9-6.6%), Kailahun 2.6% (1.6-4.1%) and Kono 2.4% (1.6-3.6%). No mf was found in persons examined in the UWA and Pujehun.

The overall arithmetic mean mf density was 50.30 mf/ml (95% CI: 39.89-60.71 mf/ml) among mf-positive individuals, and 1.19 mf/ml (95% CI: 0.90-1.48 mf/ml) in the population examined (Table 1). There was significantly higher mf density in the male population than in the female population (p < 0.0001). There was also a significant difference in mf density among age groups in the total population examined (p = 0.041). There was no significant difference in mf density by sex or age groups of infected persons (p > 0.1).

### Spatial prediction of LF distribution

**Figure 3:**
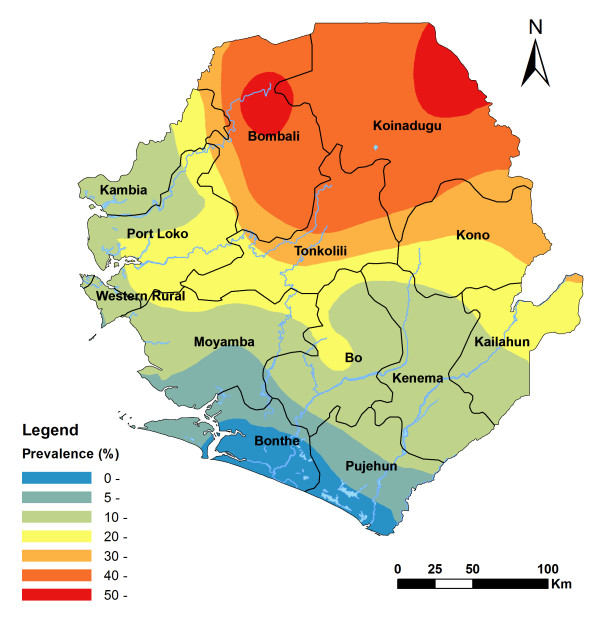
**Spatially smoothed contour map of predicted LF prevalence by ICT cards in Sierra Leone**.

**Figure 4:**
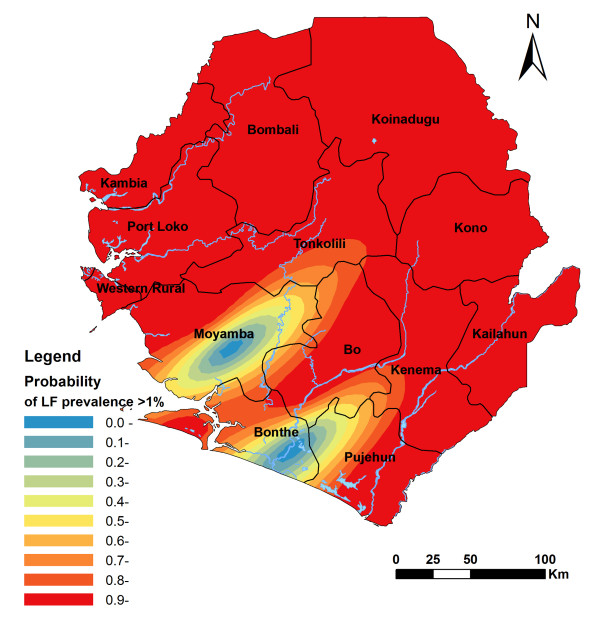
**Spatially smoothed contour map of the predicted probability that the prevalence of LF antigenaemia exceeds 1%**.

The spatial analysis of the ICT card data showed a strong spatial correlation pattern as the semi-variance in prevalence data in relation to the distance between survey sites (Figure not shown). The predicted spatial distribution of LF by kriging is shown in Figure 3. This shows a widespread distribution of LF prevalence with a clear geographical distribution pattern in Sierra Leone: high (> 40%) in a large area spanning the northeast of the country with two clusters of predicted prevalence of over 50%, gradually decreasing towards the southwest, and ending low (< 5%) in the coastal part of Bonthe and Pujehun districts. Figure 4 shows the predicted probability of the LF prevalence being over 1%, which shows high probability throughout the country with only two small clusters of relatively low probability (< 50%) in Bonthe and Moyamba.

## Discussion

All districts in Sierra Leone were endemic with LF and qualified for MDA. Distribution of LF prevalence showed a strong spatial correlation pattern with high prevalence in a large area in the northeast gradually decreasing to relatively low prevalence in the southwest coast. ICT results showed two distinct patterns: males were more infected than females and districts in the northeast part of the country had a higher prevalence than other districts. The ICT results obtained in this study were higher than in 2004 but the same pattern of a higher prevalence for circulating filarial antigen in the north than all other regions was repeated [22]. Three distinct patterns were also noted in the microfilaraemia survey: microfilaraemia was higher in males (3.0%) versus females (1.8%), increased with age in the population peaking at 41-50 years, and showed higher prevalence in the northeast than in other parts of the country. In two districts (UWA and Pujehun), microfilaraemia was not identified among the subjects examined, even though ICT prevalence was 11.7% and 4.4% respectively.

The mf prevalence was lower than that reported in 1996 but that study was conducted in an area with clearly visible signs of the disease to highlight the seriousness of the problem [21]. There were many such areas in the districts where LF signs were clearly visible among the population but in this study the sites were randomly selected to avoid bias, therefore the results of this study were more representative of the mf prevalence in the district population. The results acquired in this study on mf prevalence and mf density/intensity will form the basis for monitoring and evaluation of the effectiveness of MDA in interrupting LF transmission in each district.

Similar patterns are noted for both studies in geographical and sex distribution of the disease, which further strengthens the notion that these results are representative of the actual national picture. The ICT positive prevalence was nine times greater than the mf prevalence. Several authors have reported that ICT positive prevalence, which detects antigen released by adult *W. bancrofti*, can be 3-5 times higher than mf prevalence. People can be infected with the disease and still be amicrofilaraemic, which may explain the zero mf prevalence in UWA and Pujehun district in these studies [27,33,34].

Previous studies demonstrated the impact of long-term treatment with ivermectin alone for onchocerciasis control on LF prevalence and transmission, which showed that in villages treated for many years with ivermectin, LF microfilaraemia prevalence and intensity were significantly lower than in untreated villages [35,36]. Antigenaemia rates were significantly higher than microfilaraemia rates generally [35,36]. Onchocerciasis was endemic in 12 out of the 14 health districts in Sierra Leone [23]. Community based treatment with ivermectin for the control of onchocerciasis in Sierra Leone started in the late 1980s but did not reach full geographical coverage due to insecurity at the beginning of the civil war in 1991 and were subsequently suspended in 1994. In 2003 community-directed treatment with ivermectin (CDTI) was introduced but therapeutic coverage was low in the post conflict setting. In 2005 the National Onchocerciasis Control Program (NOCP) was reorganized and therapeutic coverage reached the prerequisite ≥ 65% and has been maintained in all endemic districts since [23]. The relatively low microfilaraemia rates in our study and the difference between antigenaemia rates and microfilaraemia rates may have been due to the ivermectin treatment for onchocerciasis control in Sierra Leone before the surveys were conducted.

The current results are in line with other studies that males have higher prevalence than females for circulating filarial antigen as well as for microfilaraemia [12,37,38]. The most probable reason for this is that males spend more time exposed to the bites of mosquitoes. The distribution of mf prevalence increasing with age shown in this study is in line with results of many other studies [9,12,33,37,38]. This emphasizes the socioeconomic impact of the disease as the age groups affected most are the major workforce in the villages. In Sierra Leone an estimated 70% of adults are farmers [39], and disability from LF incapacitates those affected and increases poverty, which is a cause for concern as the country is among the poorest in the world and demands appropriate attention for elimination of the disease. It has been suggested that adults could be more exposed to mosquito bites because of higher relative heat, more carbon dioxide output or simply because they have relatively greater surface area that can be bitten by mosquitoes [38]. Similar studies on antigenaemia and microfilaraemia for *W. bancrofti *have been conducted in other countries that reflected similar gender and age pattern as our studies [40-42].

There are certain limitations for the current studies. Firstly, children below 15 years were not selected for circulating filarial antigen and for microfilaria according to the WHO guidelines [24]. The WHO profile for LF in Sierra Leone in 2004 indicated that children were infected and that the infection could be acquired early in life [22]. It has been suggested that excluding children below 15 years could bias the studies towards older people and since it is common knowledge that filariasis infection increases with age, the prevalence might have been overestimated for the general population compared to other studies that used population based sampling methodology [33]. While this may have been true in this study, considering the overall global objective is LF elimination, such slight overestimation of LF prevalence due to the age bias should not have made much difference in terms of MDA decision in Sierra Leone. Secondly, it is recommended that ICT cards be stored at or around 8°C [13]. Although efforts were made to keep the cards in cold box during the field work, the relatively poor field conditions in the remote villages may have made it difficult to keep the box cold at all time. In such field conditions, reading every card within the time limit may not have been guaranteed. This may in part explain the higher ICT positive prevalence (nine times greater than the mf prevalence) in the current studies than in other studies [27,33,34]. However, taking both ICT and mf positive prevalence together, there is a strong correlation between the results of the two surveys for the 14 health districts as shown through correlation analysis. Therefore, the results can be considered to be representative of the true LF endemic situation in Sierra Leone.

Based on the information provided by these studies, the national NTDCP started LF MDA in 2007 [23]. Four rounds of MDA with albendazole and ivermectin have been delivered in 6 districts, three rounds in 7 districts RWA and two rounds in the UWA [43]. The mid-term impact assessment is now being conducted at the sentinel sites plus several hot spots with local knowledge of high occurrence of LF morbidity using blood smears for mf detection. It is hoped that the assessment results will provide tools to evaluate the impact of the MDA and to adjust the course of MDA if necessary.

## Conclusion

LF mapping using ICT cards was successfully conducted in 2005 in all districts of Sierra Leone which showed that all districts were endemic for LF and qualified for MDA. Baseline data collection with night blood smear was conducted in 2007-08 before MDA, which provided baseline values for mf prevalence and mf density and confirmed LF endemic status determined by ICT card survey across the country. These surveys provided tools for the NTDCP to design and implement MDA and provided the basis for future monitoring and evaluation of the national LF elimination programme.

## Competing interests

The authors declare that they have no competing interests.

## Authors' contributions

JBK was the NTDCP national programme manager, designed the studies and initial reports. MMB led and conducted the field work. MSB and MHH conducted the data entry and initial analysis. JBK and MHH drafted and revised the paper, conducted correlation analysis. MJB provided support during revision of the paper and revised the paper. YZ conducted the final data analysis, spatial analysis and revised the paper. All authors reviewed and approved the final manuscript.
